# Fully Automated Segmentation of Bladder Sac and Measurement of Detrusor Wall Thickness from Transabdominal Ultrasound Images

**DOI:** 10.3390/s20154175

**Published:** 2020-07-27

**Authors:** Zeynettin Akkus, Bae Hyung Kim, Rohit Nayak, Adriana Gregory, Azra Alizad, Mostafa Fatemi

**Affiliations:** 1 Department of Cardiology, Mayo Clinic, Rochester, MN 55905, USA; akkus.zeynettin@mayo.edu; 2 Department of Physiology and Biomedical Engineering, Mayo Clinic, Rochester, MN 55905, USA; baehyung.kim@gmail.com (B.H.K.); Alizad.Azra@mayo.edu (A.A.); 3 Department of Radiology, Mayo Clinic, Rochester, MN 55905, USA; Nayak.Rohit@mayo.edu (R.N.); Gregory.v.adriana@mayo.edu (A.G.)

**Keywords:** bladder segmentation, deep learning, detrusor muscle thickness, dynamic programming, transabdominal ultrasound

## Abstract

Ultrasound measurements of detrusor muscle thickness have been proposed as a diagnostic biomarker in patients with bladder overactivity and voiding dysfunction. In this study, we present an approach based on deep learning (DL) and dynamic programming (DP) to segment the bladder sac and measure the detrusor muscle thickness from transabdominal 2D B-mode ultrasound images. To assess the performance of our method, we compared the results of automated methods to the manually obtained reference bladder segmentations and wall thickness measurements of 80 images obtained from 11 volunteers. It takes less than a second to segment the bladder from a 2D B-mode image for the DL method. The average Dice index for the bladder segmentation is 0.93 ± 0.04 mm, and the average root-mean-square-error and standard deviation for wall thickness measurement are 0.7 ± 0.2 mm, which is comparable to the manual ground truth. The proposed fully automated and fast method could be a useful tool for segmentation and wall thickness measurement of the bladder from transabdominal B-mode images. The computation speed and accuracy of the proposed method will enable adaptive adjustment of the ultrasound focus point, and continuous assessment of the bladder wall during the filling and voiding process of the bladder.

## 1. Introduction

Bladder wall thickness (BWT) has been proposed as a biomarker for the assessment of bladder dysfunction and associated with bladder outlet obstruction (BOO) in men [1,2,3,4,5], detrusor overactivity in women [6,7,8,9], and urethral valves or abnormal urethral function in children [10,11,12,13]. The current practice to assess bladder dysfunction is cytometry analysis performed by urodynamic study (UDS), which involves the use of catheters to fill the bladder and measure the pressure detrusor pressure. Although the UDS is recognized as the gold standard for bladder dysfunction, it is expensive, time-consuming (~30 min), invasive (causing anxiety and discomfort), has a risk of urinary tract infection, can cause changes in the perception of bladder sensation, and requires physicians experienced in UDS to interpret the results. Therefore, there is an increased demand for noninvasive methods to assess bladder dysfunction. Recently, ultrasound bladder vibrometry (UBV) has been developed to noninvasively measure the mechanical properties of the bladder [14,15,16]. UBV utilizes an ultrasound array transducer to generate acoustic radiation force that excites transient waves in tissues and uses a Lamb wave model to extract parameters of elasticity based on standard least-square fitting procedures and wave dispersion analysis along the bladder wall. This technique allows one to measure the modulus of elasticity and the group velocity of Lamb waves for the bladder wall. BWT is also an essential parameter for calculating the modulus of elasticity of the bladder wall in UBV studies [14] as well as an important biomarker to assess the bladder dysfunction. To the best of our knowledge, there has not been an automated method that measures the BWT from transabdominal B-mode images, which is essential to measure BWT in an automated manner during the continuous assessment of bladder wall and its thickness during the voiding and filling processes.

Transabdominal ultrasound bladder imaging is noninvasive, inexpensive, nonradioactive, and widely accessible. Oelke et al. [17] compared the measurement of BWT obtained by conventional ultrasound with the automatic measurement performed by the BVM 6500 portable device by Verathon^®^ (formerly Diagnostic Ultrasound^®^; Bothell, WA, USA). The 3.7 MHz scanner of BVM 6500 is positioned on the patient’s lower abdomen, and the scanned images of a bladder are transmitted to a server computer via the internet, where the bladder is delineated, and BWT is measured. It takes approximately 2 min to process the images and send the BWT result back to the operator. Although both methods show good reproducibility, the conventional measurement showed the smallest variation and higher reliability. The portable BVM 6500 device also cannot accurately measure bladder thickness above 4 mm. Farag and Heesakkers [18] and Kuhn et al. [7] compared the various pathways of ultrasound to measure BWT, and they concluded that the study of BWT by the transvaginal transducer is more appropriate in women.

**Figure 1:**
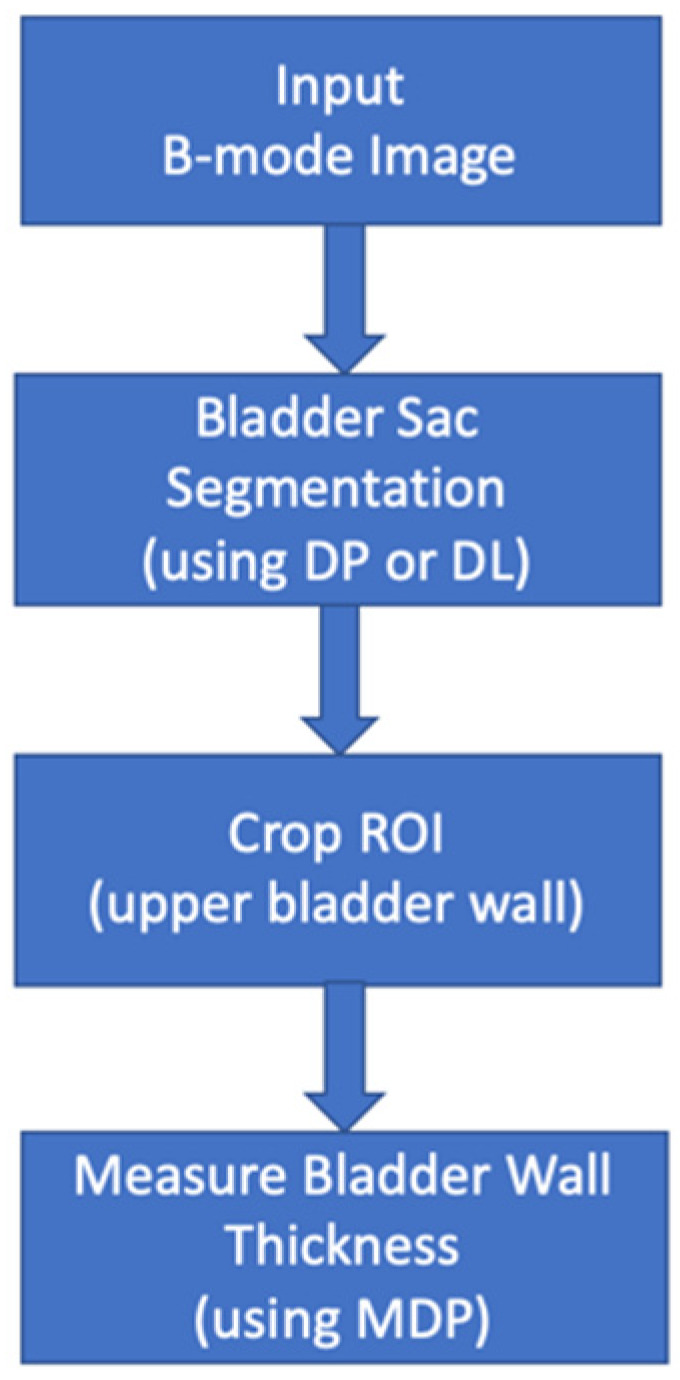
The schematic illustration of the automated processing pipeline.

In this study, we propose a two-step approach, based on deep learning (DL) and dynamic programming (DP), to estimate BWT in a fully automated manner. The first step includes accurate segmentation of the bladder from transabdominal B-mode images, and the second step includes segmenting of the anterior bladder wall using the first step output and measuring average wall thickness. A schematic illustration of the processing steps is shown in Figure 1.

## 2. Materials and Methods

### 2.1. Data Acquisition

This study was Health Insurance Portability and Accountability Act (HIPAA) compliant and was approved by the Mayo Clinic institutional review board-approved protocol (IRB application# 17-002139, date of approval 9/8/2017). A signed, written informed consent was obtained from each enrolled subject prior to the study.

We collected ultrasound B-mode images at different volumes from 11 healthy volunteers using an Alpinion E-CUBE 12R (ALPINION MEDICAL SYSTEMS Co., Ltd., Dongan-gu, Anyang-si, Gyeonggi-do, Korea) ultrasound system with a curved-linear Alpinion C1-6 probe that has 128 elements and a center frequency of 2.5 MHz. Each volunteer started with a full bladder. Data were collected at different bladder volumes as the subject voided in increments. The subject pool included a total of 5 females and 6 males. The age of the subjects varied between 22 and 71 years, with an average age of 36.18 years. The BMI of the subjects varied between 20.7 and 34.9, with an average of 26.23.

### 2.2. Bladder Segmentation

We propose two approaches to segment the bladder from transabdominal B-mode ultrasound images. The first approach is based on dynamic programming that seeks the optimal path for the continuity of the inner boundary of the bladder wall. The second approach is based on deep learning that extracts hierarchical features of the bladder to detect the bladder structure in the image. The second approach is specially developed to reduce the computation time for the segmentation task for real-time measurements of the bladder wall.

#### 2.2.1. Dynamic Programming (DP)

Dynamic programming (DP) is used to tackle several problems by continuously searching locally optimum results [19,20,21,22,23,24]. The DP technique allows one to find an optimum connective route across a graph of nodes. In the case of an image, the graph can be considered as a matrix of cost values associated with an image. The optimum route is searched across the graph of the node, which is the summation of each node on the route that is minimal. Based on preferred connectivity, a step size is chosen, which is the largest distance between two graph nodes in sequential columns.

The proposed DP-based bladder segmentation contains several steps: detecting a rough central point of the bladder; obtaining a gradient image; resampling the gradient image by casting 360 rays with an increment of 1 degree around the rough central point and putting them together in a rectangular coordinate; detecting the inner boundary with the DP; and projecting the boundary back to the original coordinate system.

**Figure 2:**
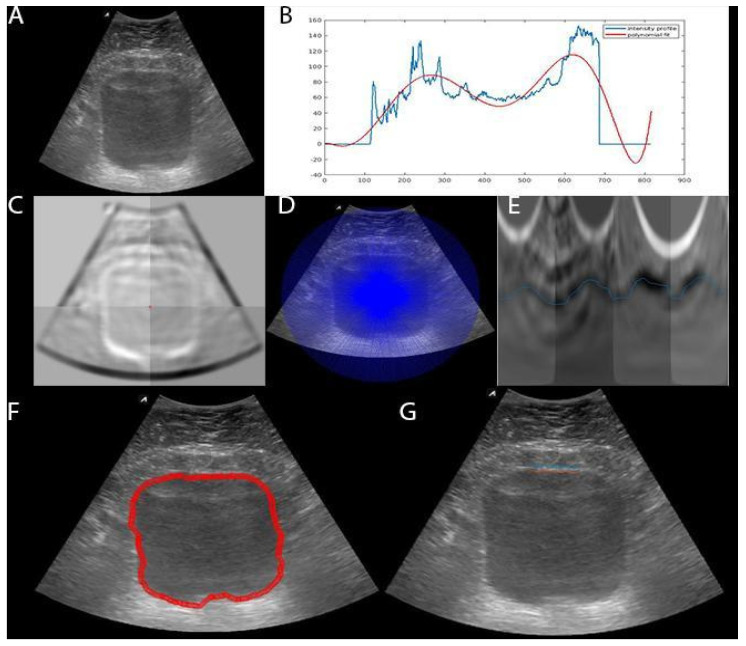
Flowchart of bladder sac segmentation using dynamic programming. (**A**) B-mode image; (**B**) Intensity profile (i.e. 0-255 gray scale) for rough centerline detection and the polynomial fit; (**C**) Gradient image; (**D**) Rays used to resample the image; (**E**) Resampled rays of inverted gradient image (i.e., reversed gradients) that dynamic programming (DP) uses to seek minimal cost pathway; (**F**) The bladder inner boundary detected in rectangular coordinate space E (blue line) transferred back to the original image space (red line); (**G**) Bladder wall segmentation using multidimensional dynamic programming. The average bladder wall thickness is 2.6 mm.

The algorithm flowchart is shown through an example in Figure 2. The input is an ultrasound image of bladder *I* with the size of width (*w*) of 1068 pixels by height (*h*) of 880 pixels. We looked at the intensity profile of an average of 15 lines (~3mm) around the center of the ultrasound probe and fitted a 4th-degree polynomial to the intensity profile to detect two peaks, representing the anterior and posterior walls of the bladder sac. The middle of these two peaks was considered as the rough center point ($c_{P}$). Next, we calculated the largest possible bladder radius, $r_{B}=w_{P}/2+|c_{P}|\times \arctan\left(fov/2\right)$, by taking into account distance from the probe to $c_{P}$, the probe width ($w_{P}=30\;\mathrm{mm}$) and the probe field of view ($fov=60^{{}^{\circ}}$), which is considered as the maximum ray length for resampling the bladder sac. Then, we resampled the gradient image that included the bladder wall boundaries along the rays around $c_{P}$ in radial 360 degrees. After that, we put all of the resampled rays together in rectangular coordinates and inverted the gradients, which is the subtraction of each gradient from the maximum gradient, to search for the optimal low-cost path. Finally, we applied DP, which minimized the cumulative cost ($\hat{C}$) function in Equation (1). This finds the optimal path for the inner boundary of the bladder that is the only continuous object in 360 degrees. After detecting the inner boundary in the rectangular coordinate, we transformed it back to the original image space.

(1) $$\hat{C}\left(w,h\right)=min\left[\hat{C}\left(w-1,h-\delta \right)+C\left(w,h\right)\times \left(1+\frac{\left|\delta \right|}{\gamma }\right)\right],$$

where C = node cost, δ=−3≤x≤3 is the step size, γ = 20 is used for soft penalization of a node cost: 5% increase for each step.

#### 2.2.2. Deep Learning (DL)

An encoder-decoder type fully convolutional neural network (CNN) model that maps the input image to the output mask for pixel-wise classification of the bladder and non-bladder tissues in ultrasound images is used. The input shape of the CNN model was the original image size of 880 × 1068 pixels. The CNN architecture that we used for this purpose is 2D U-Net [25]. The encoding part of the U-Net architecture includes 10 convolutional layers using 3 × 3 kernels and 4 max-pooling layers. The rectified linear unit (ReLU) [26] is used for the activation function. The encoding part projects the input image in low dimensional space. The decoding part of the CNN model includes 9 convolutional layers of 3 × 3 kernels and 4 upsampling layers. This part reconstructs the output predictions from the reduced low dimensional representation of the input image. We used exactly the same number of feature maps as used in U-Net architecture [25]. We used Glorot uniform distribution [27] to initialize the weights of the CNN model. At the final layer of the network, we used a softmax function to produce two-class output (i.e., bladder and non-bladder). In order to prevent overfitting, we used dropout regularization [28] in the encoding part. An Adam [29] optimizer was used to update the CNN model weights iteratively during the training with the initial learning step of 10^−5^. To reduce the error between true and prediction labels, and balance the impact of each class during the training, we used a weighted categorical cross-entropy loss, as shown in Equation (2).

(2) $$L=\frac{1}{n}\sum_{i=1}^{n}\sum_{j=1}^{m}-y_{ij}log\left({\hat{y}}_{ij}\right)w_{ij},$$

where *n* is number of samples, *m* is number of classes, y is true labels, $\hat{y}$ is predicted labels, and $w_{ij}$ is the weighting for each sample of classes. $w_{ij\;}=max\{n_{0}\ldots \;n_{j}\}/n_{j}$ is defined to balance the impact of each class in the loss function.

As we had a limited dataset for training, we used 5-fold cross-validation to evaluate the performance of the CNN and augment the training samples 10 times, using random elastic deformation.

### 2.3. Bladder Wall Thickness Measurement

**Figure 3:**
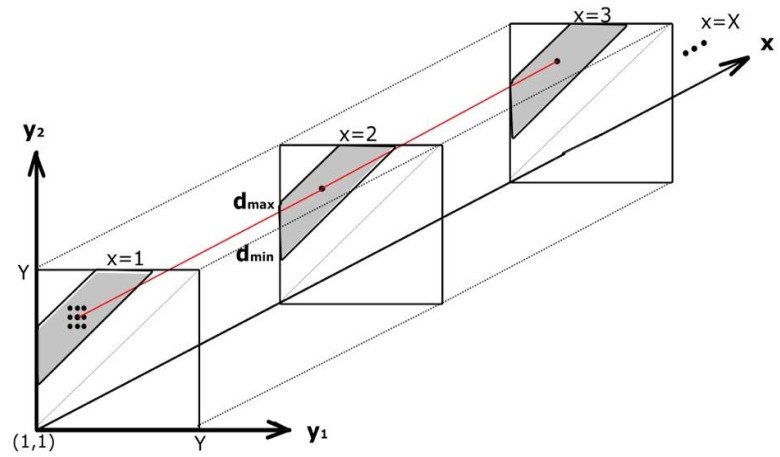
An illustration of multidimensional dynamic programming for finding optimal bladder wall boundaries. $y_{1}\;\mathrm{and}\;y_{2}$ are gradients of the cropped bounding box for each line along the column x axis. d is the distance between bladder upper and lower boundaries.

We measured the bladder wall thickness at the anterior bladder wall by using multidimensional dynamic programming (MDP) for tracking dual parallel lines [21]. The inner and outer boundary of the bladder wall are parallel, and the distance (d) in between two boundaries is $d_{min}<d<d_{max}$. $d_{min}$ and $d_{max}$ were chosen to be 1 mm and 7 mm, respectively, based on the bladder wall thickness range in adults reported in the literature [30]. As shown in Figure 3, MDP is a 3-dimensional graph that searches the optimal path of two parallel lines with a certain distance in between, while DP is a 2-dimensional graph that searches only the inner boundary of bladder sac. We limited our search in the MDP graph, shown in Figure 3, to the gray zone, which implied the inner and outer boundaries location with the distance limits. As we detected the inner boundary in the bladder segmentation step, we used this prior information to narrow our search field for the bladder wall. We only searched for the wall boundaries in a 6 mm × 14 mm bounding box (cI) in size of Y-by-X around the mid-top of the inner boundary. The search field for the bladder wall boundaries is 8 mm above and 6mm below the average y position of the inner boundary in 6mm width around the mid-x point of the bladder. The values of the search field are defined based on bladder thickness observed in our dataset, which is also higher than the upper limit of bladder wall thickness (~5 mm) reported in the literature. We used MDP to minimize the cost in equation 3 in order to find the optimal two parallel lines along the bladder wall. The node cost $y=y_{1}+y_{2}$ shown in Figure 3 was calculated by summing up the gradient of each line of bounding box at each x column point ($y_{1}$ = ∇cI is the gradient of the cropped search field, $y_{2}$ = $\bar{\nabla cI}$ is inverted gradient of $y_{1}$).

(3) $$\hat{C}\left(x,y_{1},y_{2}\right)=min[\hat{C}\left(x-1,y_{1}-\delta_{1},y_{2}-\delta_{2}\right)+C\left(x,y_{1},y_{2}\right)\times {\left(1+\alpha_{1}\right)}^{\left|\delta_{1}\right|}\times {\left(1+\alpha_{2}\right)}^{\left|\delta_{2}\right|}\times \;\left(1+\alpha_{3}\left|\left(y_{1}-y_{2}\right)-\left(\delta_{1}-\delta_{2}\right)\right|\right),$$

where $\delta_{1}$ and $\delta_{2}$ are the step size in x and y coordinates, $\alpha_{1}$ and $\alpha_{2}$ are the step size penalty for *x* and *y*, $\alpha_{3}$ is the distance penalty between two parallel lines. $\hat{C}$ and C are cumulative cost and node cost, respectively.

$\delta_{1}=1\;pixel$ and $\delta_{2}=1\;pixel$ were selected as there was continuity in the bladder wall, and it was not expected to be a displacement of more than 1 pixel in each direction. $\alpha_{1}=0.2$ and $\alpha_{2}=0.2$ were empirically chosen to be a 20% increase of the node cost, which was a soft continuity penalty. $\alpha_{3}=0.2$ was selected to be a 20% increase of node cost to force the distance between two lines to be constant (parallel line continuity). The penalty applied to the node cost should not be too hard or too soft. A too-hard penalty would dominate the overall cost and not pick the smooth boundaries, while the too soft would have no impact on the overall cost. We empirically adjusted penalties until we observed the consistency in measurements, as suggested in [19]. We observed that a 20% node cost increase was a satisfactory penalty to find an optimal solution.

### 2.4. Evaluations

Three independent observers manually segmented the bladder and measured the bladder wall thickness. We compared the performance of DP and DL methods to the manual reference of the three observers and measured interobserver variability by calculating the Dice similarity index that provides the overlap between two given segmentations. As the manual segmentation is a laborious task, we only segmented a subset of images (46 images out of 80 images of 11 subjects) to reduce the redundancy. We evaluated the performance of the DL segmentation using 5-fold cross-validation and the data were split based on the subject level.

To assess the performance of MDP for Bladder Wall Thickness (BWT) measurements, we compared the performance of our fully automated method to the average wall thickness obtained from the three observers manual wall thickness measurements. The root-mean-square error (RMSE) calculated over 80 images obtained from 11 subjects. To calculate the inter-observer variability, three observers measured the BWT and the average of these three measurements was calculated; then, the BWT measured by each observer was compared to the calculated averaged measurement. The Bland–Altman analysis was used to show the agreement between the automated method and manual reference, and between the three observers.

We used an HP Z820 workstation with Intel Xeon E5-2640 2.5 GHz processor and an NVIDIA Titan X Graphical Processing Unit (GPU) card that has 12 GB memory for processing the data. The deep learning model was implemented with the Tensorflow package.

## 3. Results

**Figure 4:**
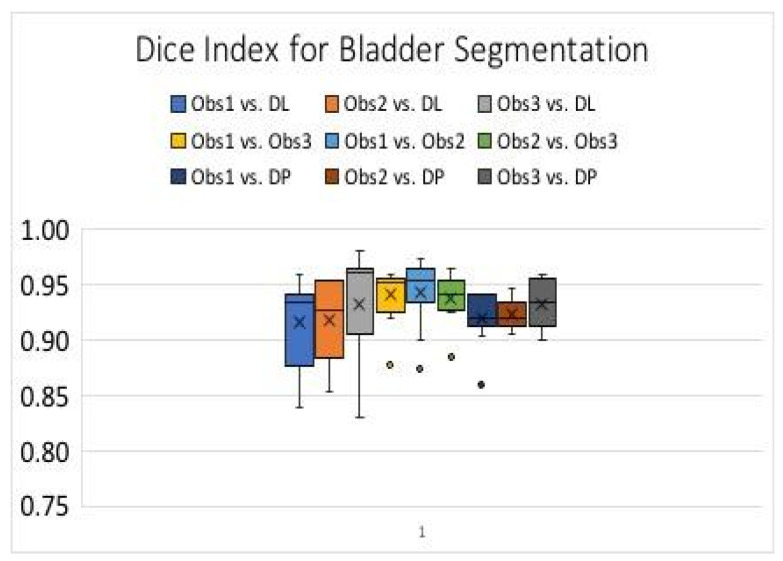
Box plot for Dice index for Bladder sac segmentation Obs: Observer, DL: Deep Learning, DP: Dynamic Programming.

**Figure 5:**
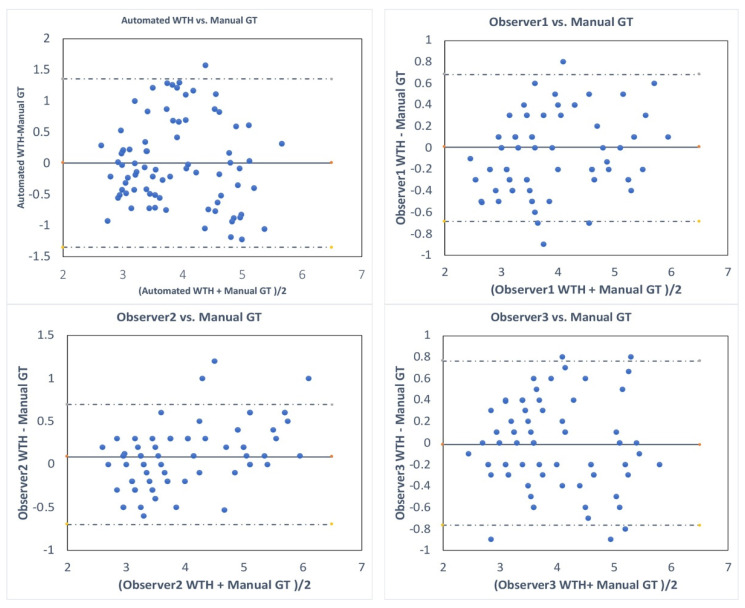
Bland–Altman plots that show the difference between automated wall thickness (WTH) measurements (blue dots) and the average manual wall thickness measurements (GT) by three observers (Automated vs. GT) and the inter-observer variability (i.e., observer 1 vs. manual GT, observer 2 vs. manual GT, and observer 3 vs. manual GT). GT: Ground truth.

Figure 4 shows the Dice index over 46 images of 11 subjects for the automated segmentation of the bladder sac, using DL and DP methods and manual segmentations by three observers. The median and mean Dice indices for the automated and manual methods are comparable and above 0.9, which is an acceptable performance for bladder sac segmentation in B-mode images that contain missing boundaries and clutter noise. The average and standard deviation of Hausdorff distance in DP vs. Observer 1 manual segmentation, DL vs. Observer 1 manual segmentation, and DP vs. DL are 4.3 mm ± 2.9 mm, 5.3 mm ± 4.4mm, and 4.9 mm ± 3.1 mm, respectively. Figure 5 shows the Bland–Altman plot for the bladder wall thickness measurements by the automated MDP method and manual ground truth. The mean of the measurements is centered at 0 difference, and all of the measurements, apart from one outlier, are scattered in 95 percentile confidence intervals. Figure 5 also shows the Bland–Altman plot for intra-observer variability. The error range between each observer manual measurement and the average manual ground truth is in the same order and slightly smaller than the error range between the automated measurement by MDP and the average manual ground truth. 

Computation time: It takes about one minute for the dynamic programming approach described in Section 2.2.1. to segment the bladder sac from a transabdominal ultrasound image. For the deep learning approach explained in Section 2.2.2. it takes less than a second to segment the bladder sac from a transabdominal ultrasound image. To measure bladder wall thickness with the MDP approach takes less than 50 ms.

The average root-mean-square-error (RMSE) and standard deviation (SD) between the wall thickness measurements of automated MDP and the ground truth is 0.7 mm ± 0.21 mm. The RMSE between three observers is shown in Table 1. The error between the automated method and the ground truth and the variability between observers are in the same order.

## 4. Discussion

Detrusor wall thickness is an important diagnostic biomarker for assessing bladder health and function. In this study, we propose a robust and automatic technique for estimation of detrusor wall thickness from noninvasive 2D ultrasound images using dynamic programming and deep-learning. To evaluate its efficacy in vivo, we tested this technique on 11 human volunteers and compared the results with manual sonographic measurements.

DP algorithms allow for the effective determination of the optimal connective path through a graph of nodes. In this study, we used a multidimensional DP method for tracking dual parallel lines that allowed an estimation of the bladder wall thickness. Further, the deep learning model involved an encoder-decoder type convolutional neural network. Using a U-Net CNN architecture, the input US sonogram was converted into a binary mask for pixel-wise classification into the bladder and non-bladder regions. The results demonstrate that bladder wall thickness estimated using multidimensional DP were accurate up to 0.7 mm ± 0.21 mm, with respect to the ground truth. Further, these results were qualitatively and quantitatively comparable to the standard deviation observed for manual segmentation across multiple observers and iterations. As shown in Table 1 and Figure 5, the automated method for measuring bladder wall thickness performs almost as well as the manual method with a slight difference (~0.2 mm = 1 pixel), compared to the variability between three observers. This variation is quite acceptable, as the manual measurements are affected by the precision of mouse clicking and clutter noise around the bladder wall.

As reported in Figure 4, the deep learning approach provides the highest median Dice index for bladder sac segmentation. The results of dynamic programming and deep learning methods, and the manual methods, are comparable to each other. Since the computation time for the deep learning approach is significantly shorter than that of the dynamic programming approach, it is preferred to be used for continuous measurements during the filling and voiding process of the bladder. Furthermore, the performance of the deep learning method could be further improved by increasing the number of training images.

A critical aspect of clinical translation of the proposed technique for reliable diagnostic use is its computational efficiency for real-time implementation. With the proposed technique, bladder wall thickness can be estimated in <1 s. For continuous assessment of the bladder wall and its thickness during the voiding and filling processes, a computation speed of the algorithm less than a second is necessary. To the best of our knowledge, this is the first study to present a fully automated method for the measurement of bladder wall thickness from transabdominal B-mode images. The only other study that attempts to measure bladder wall thickness automatically [16] used a customized device and could only measure BWT up to 4 mm. As presented in their study, it takes about 2 min to process the scanned images for measuring BWT, which makes it unsuitable for the continuous assessment of BWT.

Image quality plays an important role in obtaining accurate manual and automated measurements. Image quality may vary depending on a bladder’s depth, which may change with the bladder volume and bladder position within the abdomen. The peritoneum, the serous membrane forming the lining of the abdominal cavity, lays on top of the bladder detrusor muscle and can sometimes make the measurement of the bladder wall thickness difficult. It is important to adjust the probe position and obtain a bladder view that gives a clear view of the bladder wall for accurate measurements.

The proposed approach implemented on a clinical ultrasound scanner can be used in various applications, such as monitoring the bladder wall thickness as a biomarker for bladder overactivity, bladder dysfunction, and outlet obstruction. A key advantage of this method is its ability to capture the dynamic changes in bladder wall thickness due to various events. As such, it is conceivable to measure bladder wall thickness changes, due to transient contractions by this method. This technique is particularly suitable for the UBV studies, which requires measuring bladder wall thickness for the assessment of bladder elasticity during the filling and voiding phases.

One of the limitations of the current study is that the proposed technique was evaluated on a limited population of healthy volunteers, and only with ultrasound vendor data. The DL model could be easily further trained on a larger dataset from multi-vendors by using transfer learning, which is to apply the knowledge learned from this dataset to a larger dataset. As shown in Figure 5, we have bladder wall thickness larger than 5 mm, which is the reported maximum bladder wall thickness in the literature. This is probably due to the thickened wall and the degradation of image quality in small bladder volumes during the voiding process. The results obtained from this pilot study are encouraging for further clinical validation of the method on patients with bladder dysfunction. Additionally, in our future work, we will evaluate the efficacy of using the proposed wall thickness estimation technique with UBV for accurately estimating bladder wall elasticity continuously.

## 5. Conclusions

In summary, we developed and presented a fully-automated and accurate method based on deep learning and dynamic programming to segment the bladder sac and measure bladder wall thickness in a time-efficient manner. The proposed method based on deep learning significantly reduces the image processing time and makes the continuous assessment of bladder activity feasible. This method presented in this paper may be used as a part of the UBV technique for applications in continuous assessment of bladder activity.

## Figures and Tables

**Table 1:** The average root-mean-square-error (RMSE) ± standard deviation (SD) for wall thickness measurements.

BWT	RMSE (mm)
MDP vs. GT	0.7 ± 0.21
Obs1 vs. Obs2	0.55 ± 0.21
Obs1 vs. Obs3	0.63 ± 0.27
Obs2 vs. Obs3	0.69 ± 0.25

MDP: Multidimensional dynamic programming. GT: Ground truth. Obs = Observer.
